# Three-Dimensional cryoEM Reconstruction of Native LDL Particles to 16Å Resolution at Physiological Body Temperature

**DOI:** 10.1371/journal.pone.0018841

**Published:** 2011-05-09

**Authors:** Vibhor Kumar, Sarah J. Butcher, Katariina Öörni, Peter Engelhardt, Jukka Heikkonen, Kimmo Kaski, Mika Ala-Korpela, Petri T. Kovanen

**Affiliations:** 1 Department of Biomedical Engineering and Computational Science, School of Science and Technology, Centre of Excellence in Computational Complex Systems Research, Aalto University Aalto, Finland; 2 Computational and Mathematical Biology, Genome Institute of Singapore, A*STAR, Singapore; 3 Institute of Biotechnology, University of Helsinki, Helsinki, Finland; 4 Wihuri Research Institute, Kalliolinnantie 4, Helsinki, Finland; 5 Department of Pathology, Haartman Institute, Haartmaninkatu 3, University of Helsinki, Helsinki, Finland; 6 Department of Applied Physics, Nanomicroscopy Center, School of Science and Technology, Puumiehenkuja 2, Aalto University, Espoo, Finland; 7 Department of Information Technology, University of Turku, Turku, Finland; 8 Computational Medicine Research Group, Institute of Clinical Medicine, Faculty of Medicine, Biocenter Oulu, University of Oulu, Oulu, Finland; 9 Department of Internal Medicine and Biocenter Oulu, Clinical Research Center, University of Oulu, Oulu, Finland; Heart Center Munich, Germany

## Abstract

**Background:**

Low-density lipoprotein (LDL) particles, the major carriers of cholesterol in the human circulation, have a key role in cholesterol physiology and in the development of atherosclerosis. The most prominent structural components in LDL are the core-forming cholesteryl esters (CE) and the particle-encircling single copy of a huge, non-exchangeable protein, the apolipoprotein B-100 (apoB-100). The shape of native LDL particles and the conformation of native apoB-100 on the particles remain incompletely characterized at the physiological human body temperature (37°C).

**Methodology/Principal Findings:**

To study native LDL particles, we applied cryo-electron microscopy to calculate 3D reconstructions of LDL particles in their hydrated state. Images of the particles vitrified at 6°C and 37°C resulted in reconstructions at ∼16 Å resolution at both temperatures. 3D variance map analysis revealed rigid and flexible domains of lipids and apoB-100 at both temperatures. The reconstructions showed less variability at 6°C than at 37°C, which reflected increased order of the core CE molecules, rather than decreased mobility of the apoB-100. Compact molecular packing of the core and order in a lipid-binding domain of apoB-100 were observed at 6°C, but not at 37°C. At 37°C we were able to highlight features in the LDL particles that are not clearly separable in 3D maps at 6°C. Segmentation of apoB-100 density, fitting of lipovitellin X-ray structure, and antibody mapping, jointly revealed the approximate locations of the individual domains of apoB-100 on the surface of native LDL particles.

**Conclusions/Significance:**

Our study provides molecular background for further understanding of the link between structure and function of native LDL particles at physiological body temperature.

## Introduction

Low-density lipoprotein (LDL) particles are specialized lipid transport vehicles in the blood. They are formed in the circulation during an endogenous metabolic cascade of apolipoprotein B-100 (apoB-100)-containing lipoproteins [1]. This cascade originates in the hepatic secretion of very-low-density lipoprotein (VLDL) particles, then proceeds as a sequential metabolic continuum in the blood, where lipoprotein particle transformations are mediated by the actions of various lipolytic enzymes and lipid transfer proteins, and reaches its completion by generation of LDL particles. By providing cholesterol to peripheral tissues, the LDL particles are the key components in physiological cholesterol metabolism [1], [2]. Hepatic LDL receptors remove LDL particles from the circulation, so tending to ensure that the concentration of circulating LDL particles remains at a physiologically relevant level [1]. However, elevated blood plasma concentrations of the LDL particles, whether of genetic or environmental origin, will attenuate the functioning of the LDL receptor pathway and enhance the influx of LDL particles into the arterial wall where the particles become trapped, modified, and thereby are converted to initiators and major players in the vicious circle of inflammation and lipid accumulation characteristic of atherogenesis [3], [4], [5]. Thus, LDL particles function at the interface between physiological and pathophysiological pathways of lipoprotein and lipid metabolism [6], [7].

All lipoprotein particles share a common structure as micellar complexes with an amphipathic surface monolayer and a hydrophobic lipid core [4], [6], [8], [9], [10]. Importantly, lipoprotein particles are biologically functional only in their native state in an aqueous environment. LDL particles consist of a single copy of an apoB-100 molecule and ∼3,000 individual lipid molecules, some present on the surface and some in the core of the particle. Of the LDL lipids, the most abundant and structurally most important are the ∼1,600 cholesteryl ester (CE) molecules present in the core of each particle [4], [8], [9], [10]. The oily lipid core is surrounded by a monolayer of phospholipids composed mainly of phosphatidylcholine and sphingomyelin, and of unesterified cholesterol molecules. The apoB-100, again, wraps around the surface of the LDL particle. It interacts with a fraction of the surface lipids, partially penetrates the phospholipid monolayer, and so may reach the outer core of the particle and interact with the lipids of this deeper layer of LDL as well [4], [9].

The LDL particles, like other lipoprotein particles, form a rather heterogeneous group of particles in that, for example, they vary in both diameter (∼18–25 nm, mean 22 nm) and density (∼1.019–1.063 g/ml). The heterogeneity of circulating LDL particles in the living body (at 37°C) reflects their dynamic state in the catabolic cascade of the circulating apoB-100 containing lipoproproteins, their structure and physical properties depending on their lipid composition as well as on the conformation of the apoB-100. ApoB-100 is a non-exchangeable apolipoprotein and one of the largest monomeric proteins known, consisting of 4,536 amino acid residues (∼550 kDa) [11]. In addition to interacting with the LDL receptors and the negatively charged glycosaminoglycans of the arterial wall, the apoB-100 molecule has a particular role in maintaining the structural integrity of the LDL particles and controlling the structural and compositional changes taking place in them [7]. ApoB-100 is understood to consist of five alternating α-helical and β-sheet domains, i.e., it is a pentapartite NH- βα1-β1-α2-β2-α3-COOH polypeptide [4], [9], [10]. The first 1000 N-terminal residues of apoB-100 have 20.1% identity and 39.6% similarity to lamprey lipovitellin (residues 1-1074) for which the atomic crystal structure is known [12]. Studies on model systems have recently suggested that the β-strand regions anchor the apoB-100 on the lipid surface whereas the α -helical domains may desorb from and reabsorb onto the particle surface [13], [14]. Hence it has been concluded that both α-helical and β-strand regions contribute to the conformational flexibility of the particle [14].

The inherent heterogeneity of LDL particle size and composition, together with the structural issues related to the architectural flexibility of apoB-100, pose serious challenges to studies aimed at elucidating structural details of the native particle. Small-angle X-ray scattering has been used to model the LDL core to low-resolution, positioning the radial extent of the lipids and apoB-100 [15]. Several groups have published electron microscopy data of raw or two-dimensionally averaged images of LDL particles at temperatures of 4°C, 10°C, 24°C, 40°C and 42°C [16], [17], [18], [19], [20]. Previously, three-dimensional (3D) reconstructions have only been reported for LDL particles at non-physiological temperatures. Orlova et al. [21] first applied electron-microscopy and image processing methods to build a 3D model of LDL at 4°C, with a resolution of 27 Å [17], [21]. Subsequently, Poulos [22] studied LDL bound to five different antibodies at 4°C to a resolution of 25 Å. Recently, Ren et al. [23] published a 28-Å resolution reconstruction of LDL bound to the LDL-receptor at 4°C. The 3D models at low temperature have revealed a core consisting of inner lamellae-like layers of lower-density, and an outer shell of higher-density with a knob-shaped protrusion. A study using small angle neutron scattering of lipid-free apoB-100 described the modular nature of the protein, with ordered domains connected by flexible linkers [24]. However, a high resolution 3D structure of the LDL particle in a native condition and temperature would help to confirm previous observations and link them to biological functions.

In the current study, we wanted to gain insight into the overall structural characteristics of LDL particles and the folding of apoB-100 under native conditions, i.e., when blood plasma-derived particles are present as lipid-apoB-100-complexes in an aqueous environment. We applied single particle reconstructions with cryo-electron microscopy images of LDL particles vitrified both at 6°C and 37°C. Due to robust filtering of noisy cryo-electron micrographs [25] and the extensive amount of individual LDL images collected, we achieved a remarkably good resolution of ∼16 Å, which provided novel insights into the molecular structure of native LDL particles with naturally folded apoB-100.

## Materials and Methods

### Ethics statement

Human plasma was obtained from healthy blood donors after informed consent as by-products from the preparation of blood products for clinical use. The study was approved by the Finnish Red Cross Blood Service (Permission number 25/2010; Finnish Red Cross Blood Service).

### Isolation of LDL particles from human plasma

Human LDL particles (density between 1.019 and 1.050 g/ml, average diameter 23±2 nm; SD; median 23 nm; range 19 – 25 nm) were isolated from the plasma of healthy blood donors (Finnish Red Cross Blood Service) by sequential ultracentrifugation in the presence of 3 mM EDTA. Briefly, solid KBr was added to plasma to adjust its density to 1.019 g/ml. Very-low- and intermediate-density lipoproteins were removed, and the density of the bottom fractions was adjusted to 1.050 g/ml with solid KBr. After ultracentrifugation for 72 h in a Type 50.2 Ti rotor (Beckman Coulter) at 30 000 rpm (g-max = 109 000 g), LDL was recovered from the top of the centrifuge tubes, re-centrifuged (density 1.060 g/ml) for 24 h in the same rotor at 40 000 rpm (g-max = 190 000 g), and dialyzed extensively against 1 mM EDTA, 150 mM NaCl, pH 7.4. The amount of LDL is expressed in terms of protein concentration, determined using the Lowry assay with bovine serum albumin as a standard [26].

### Cryo-electron microscopy

Samples at a concentration of approximately 0.3 mg protein/ml were vitrified on Quantifoil holeycarbon grids (Quantifoil Micro Tools, GmbH) either at 6°C or 37°C, allowing pre-equilibration of the sample at the desired temperature for at least 45 minutes prior to plunging [27]. Cryo-electron microscopy was carried out at 200 kV and x 50 000 magnification on an FEI Tecnai F20 field emission gun transmission electron microscope (Electron Microscopy Unit, Institute of Biotechnology, University of Helsinki, Finland). Micrographs were scanned on a Zeiss Photoscan TD scanner as described previously [28]. This resulted in digitized images with a nominal sampling of 1.4 Å per pixel. Tilt series of three micrographs were collected at a magnification of x 19 000 using zero, four and eight degree tilt angles, resulting in a sampling rate of 3.35 Å per pixel in the scanned images.

### Single particle reconstruction of LDL particles

Particles were picked from micrographs free from drift and astigmatism semi-automatically with EMAN's graphical program Boxer [29] that picks particles by cross-correlating selected template images over the whole micrograph. At 6°C 52 micrographs and at 37°C 23 micrographs were used with defocus values in the range of approximately 1.5 µm to 4.7 µm. After manual inspection, datasets of 71,521 (6°C) and 29,844 images (37°C) resulted. Initially only phase-flipping was carried out to correct for the contrast transfer function. To reduce the effect of image noise and artifacts, an initial single particle reconstruction was made on images denoised with an information theory [Minimum Description Length (MDL)] based method which we have introduced earlier [25]. Fifty initial class averages were obtained using a reference-free classification of 3000 images by multivariate statistical analysis using parameters, as suggested in EMAN [29]. Angles were assigned using the cross common lines method prior to reconstruction using weighted-back projection [30], [31]. The initial 3D models were low-pass filtered. The 2D image dataset was increased to include all of the particles and iterative refinement continued. After each iteration of the single-particle-reconstruction process only 70–75% of the images with the highest correlation values were chosen.

Refinement was done to remove images with high noise and artifacts. First reference-free classification was done for the initial sets of denoised LDL images. A radial profile of each of the LDL image-class-averages was then compared to the radial profiles of all the projections from the reconstructed 3D volume. The images belonging to the class-averages which had unacceptable radial-profile correlation (less than 0.2) with all the 3D-projections were rejected from the data set. Datasets from the two different temperatures were handled separately. Finally 67 678 and 26 083 images at 6°C and 37°C, respectively, were selected in EMAN. In order to avoid any bias in 3D reconstruction due to the denoising, the final reconstructions were made using non-denoised images after being assigned to classes using their denoised equivalents.

To address the heterogeneity remaining in the final reconstructions, after radial-profile sorting of the particles, a bootstrap method was used to calculate the 3D variance maps for both of the reconstructions [32], [33]. The selection of images for the bootstrap reconstructions was performed after classification of images and before the 3D reconstruction. For each dataset, 200 bootstrap 3D volumes were created from which both a 3D average and a 3D variance map were then calculated. At both temperatures 3D variance maps due to noise and alignment were also estimated by using the same number of images containing only background [32], [33]. A 3D variance map calculated from images of just the vitrified water background was subtracted from the 3D variance map calculated from the LDL 3D reconstruction. Thus, the resulting 3D variance should represent mainly differences due to conformation rather than background noise.

### Fitting the 3D locations of antibodies

The program ADP_EM [34] was used to dock the back-bone structure of the first 780 amino acids of lipovitellin (PDB code: 1LSH) to the 3D reconstructions [35], [36], [37]. The reconstructed 3D volume was bandpass filtered to include information between 15 and 80Å prior to fitting. Then the ADP_EM docking tool was used to fit at a resolution of 17Å and a density threshold of 1.7 standard deviations above the mean intensity [34], [38]. The P-value was calculated using the Z-score of the first 40 fitting results, of which the first 10 results are shown in Table S1. It can be clearly seen that the P-values of the first two solutions are much more significant than the other possible solutions. We tested additional threshold and resolution cutoffs, but the top results remained very similar to that reported here, even when a threshold of 0 sigma was used. The fit was tested with both possible hands of the reconstruction.

The handedness of the reconstructed volume of LDL at 37°C was confirmed to be correct by collecting tilted images at 0, 4, and 8 degrees. Both the tilt series and the fitting results were consistent with the handedness reported here. After finding the most probable location of the first 780 amino acids of lipovitellin, a 3D cartoon model was made to represent the probable locations of monoclonal antibodies using coordinates of latitude and longitude that have been described earlier [39]. The alignment of the cartoon model to the LDL 3D reconstruction needed three anchoring points. For the first two locations, we chose antibodies MB19 and MB24, which are located in the docked lipovitellin-like structure. For the third point we used antibody location MB11, and placed it close to the C-terminus of the docked PDB structure. MB11 represents apoB-100 amino acids 995–1082, and we chose its location with respect to the docked lipovitellin structure, as suggested and modeled by Richardson and coworkers [40].

## Results

**Figure 1:**
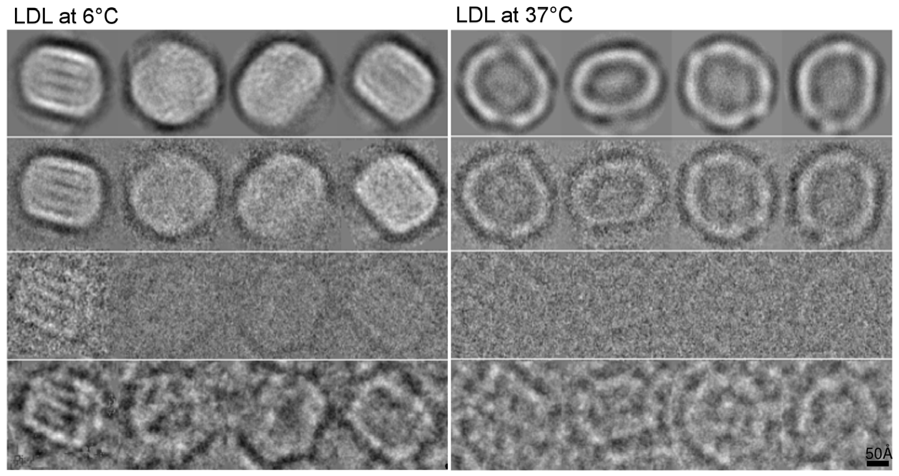
Comparison of raw and averaged data for LDL particles at 6°C and at 37°C. The first rows show projections from the reconstructed volume in different views, the second rows show the corresponding class averages. The third rows show one of the original images from each class average and the bottom rows show the corresponding denoised image.

The 3D reconstructions of the LDL particles in this study were derived solely on the basis of the acquired data, i.e., without any prior knowledge on the LDL structure or chemical composition of the LDL preparations isolated by ultracentrifugation. Careful gradient fractionation and selection *in silico* ensured that most of the LDL particles in the sample were approximately of the same size (**Figure S1**). We found that the correlation of the radial profiles of different class averages, which were obtained by comparing reference-free classification to model projections in the same orientation, was a useful selection criterion to decrease size heterogeneity. We used it to eliminate ∼5% of the 6°C data and ∼13% of the 37°C data with artifacts and projections from other size LDLs belonging to minority groups. The correspondence between typical raw data, denoised images, class averages and projections of the final model are shown in **Figure 1**. We achieved a resolution of ∼16 Å for the final reconstructions of LDL at both temperatures using a Fourier Shell Correlation cutoff of 0.5 [41] (**Figure S2**). In general, our data and the particle models derived agree well with those published previously at lower resolution at 4^o^C [21].

### Structural details and shape of LDL particles

**Figure 2:**
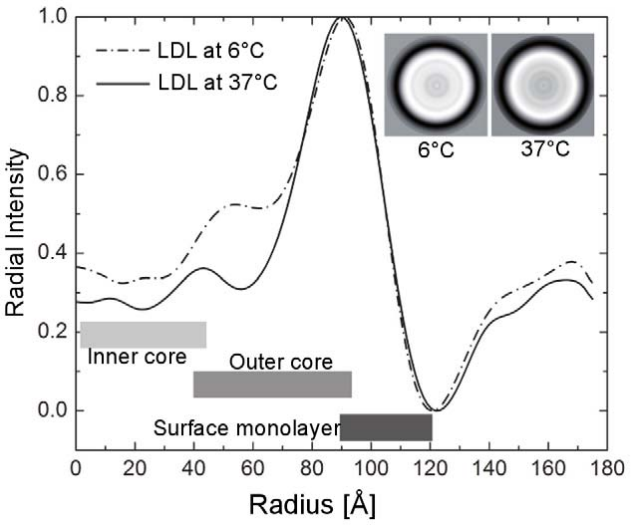
Scaled radial intensity profiles of the reconstructed volumes of LDL particles. The overall intensity of the interior of the LDL particle reconstruction is higher at 6°C than at 37°C. This indicates a pseudo-random structure of lipids in the LDL core at 37°C, while at 6°C the organization is more rigid. The 2D averages are shown in the insets.

**Figure 3:**
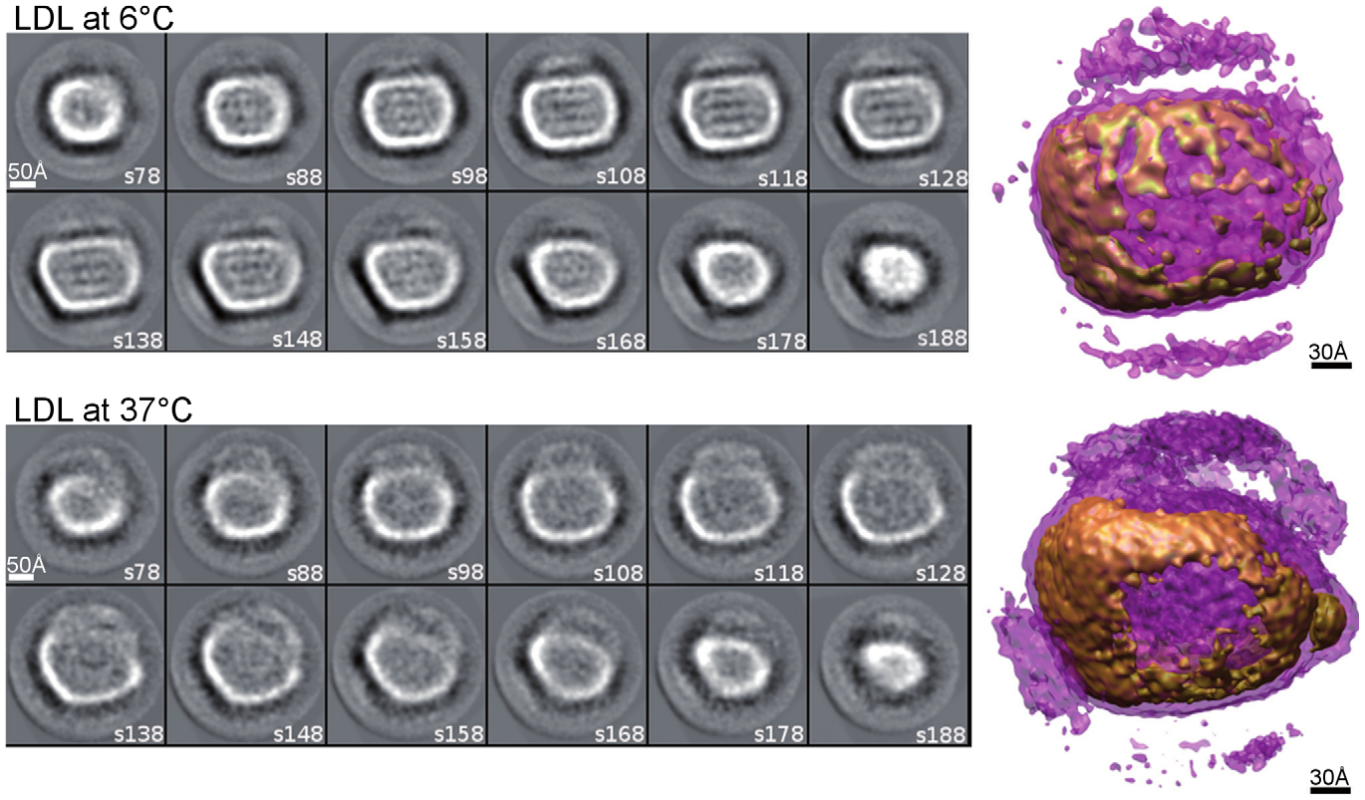
Consecutive sections through the 3D reconstructions of LDL particles at 6°C and at 37°C. Each slice is as thick as a pixel whose size is 1.4 Å. The slice number is shown in the bottom right corner of each image. The slice with number s128 is the central slice of the 3D volume, while s78 marks the slice which is 50 pixels (70 Å) behind the central slice. Similarly s188 marks the slice which is 60 pixels (84 Å) in front of the central slice. The transparent violet isosurfaces for the corresponding 3D maps are made at threshold of µ+0.7σ (showing mainly the lipid surface of the particles) and the opaque brown high-density isosurfaces are made at threshold of µ+3σ (showing mainly rigid structures of apoB-100). These 3D reconstructions were made in the final iterations of the single particle reconstructions using non-filtered 2D cryo-EM images after determining the classes of their filtered versions.

It became evident already from the micrographs, that the LDL particles vitrified at 6°C were more organized than those vitrified at 37°C (**Figure 1**), and that this applied particularly to the core of the particles, as shown by the radial density profiles of the reconstructions in **Figure 2**. The overall intensity of the core region appears clearly higher at 6°C than at 37°C up to a radius of approximately 75 Å. The inner core intensity peaks for LDL particles at 6°C are distinctly visualized in the sequential cross-sections through the models shown in **Figure 3**, and were interpreted to present planar cholesteryl ester layers. Importantly, these layers are approximately 30Å apart which strikingly agrees with the organization of the cholesteryl ester molecules in a smectic liquid crystal-like phase [41]; i.e., the cholesterol ring structures of the CE molecules correspond to the denser areas and the fatty acid tails correspond to the less dense intervening regions (**Figure 3**). As indicated by the lower radial intensity in the inner and outer core regions of the particle, there is significantly less ordering inside the particle at 37°C than at 6°C (**Figure 2**). Thus, at the physiological temperature of 37°C, the core CE molecules in LDL particles appear to be in a liquid-like state. The radial intensity curves show a similar hydrodynamic radius for the LDL particles both at 6°C and at 37°C suggesting that, in contrast to the physical reorganization of the lipids, the conformation of apoB-100 on particle surface, experiences only minor temperature-related changes within the studied temperature interval.

**Figure 4:**
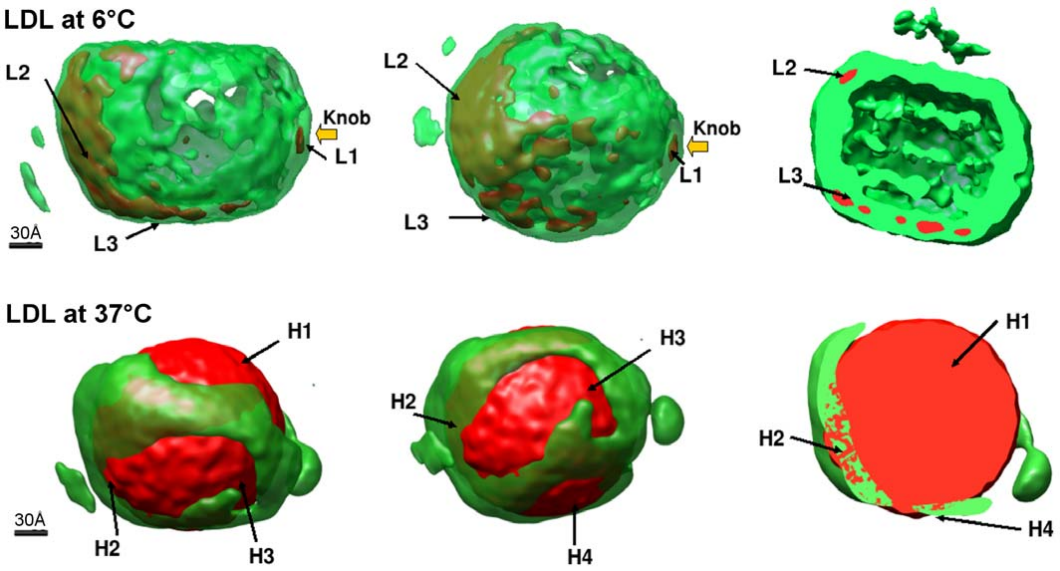
Main regions of variance within the LDL particle reconstructions. A boot-strap method was used to produce three dimensional variance maps (red) superimposed on average maps (green) of the 6°C and 37°C particles. The average volume is shown at threshold of µ+2σ intensity level. The regions of high variance for LDL at 6°C are labeled as L1, L2, and L3 and for LDL at 37°C as H1, H2, H3 and H4. For the rightmost illustration at 6°C the threshold for the average map was lower (at µ+σ intensity level) and the volume was cut in the middle to visualize the inner core structure.

The reliability and consequent interpretation of the final reconstructions could have been affected by heterogeneity in the LDL particles, for instance in conformational differences in apoB-100, as well as by errors arising from incorrect assignment of angles to individual particles. Hence, the particles were selected by physical separation prior to vitrification and by radial-profiling to remove outliers, and finally 3D variance maps were calculated to determine which features in the reconstructions were most reliable. We applied a bootstrap method [33] using 200 bootstrap 3D volumes of LDL particles both at 6°C and 37°C. The maps are shown together with the 3D average maps in **Figure 4**. At 6°C there is less variance in the center of the particles than at 37°C, reflecting the more ordered smectic phase of the CE molecules at 6°C in comparison to the liquid-like organization at the physiological temperature. It is noticeable that the majority of the most variable sites are associated with the lipid core at 37°C, except H2 (**Figure 4**). The high variance regions H1, H3 and H4 in LDL at 37°C in the inner shells are probably due to the fluidity of the lipids, but H2 could be due to the flexibility of lipid-associating domains of apoB-100. Similar variability was seen at 6°C (**Figures 4**, region L2 versus **Figures 4**, region H2 at 37°C) which could be due to protein conformational changes rather than lipid. There is also a high variance region L1 in LDL at 6°C which corresponds to the lipid-free knob in the 37°C LDL volume (**Figure 4**).

### Secondary structure of native apoB-100

**Figure 5:**
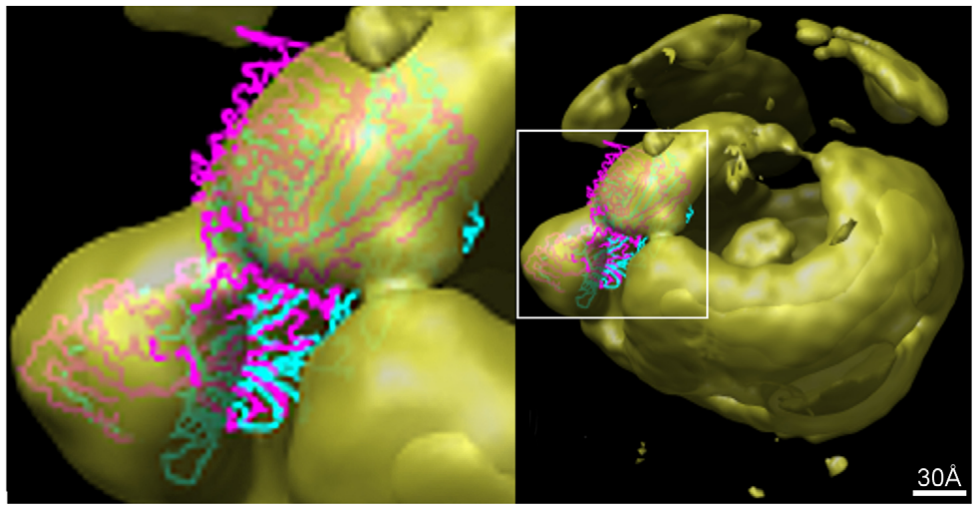
Results of the fitting of the backbone of lipovitellin PDB structure (PDB code: 1LSH) with the LDL reconstruction at 37°C. The isosurface shown here is from a 3D volume which has been low pass filtered to 17 Å after thresholding at 1.7σ above the mean intensity. The PDB structure of the backbone has been shown with different colors representing their rank assigned by the docking tool ADP_EM. The magenta color is for rank 1 and cyan for rank 2. Both of the two best results are docked similarly although there is slight rotational difference between the best and second-best docked results. The boxed lipovitellin region is shown enlarged on the left.

**Figure 6:**
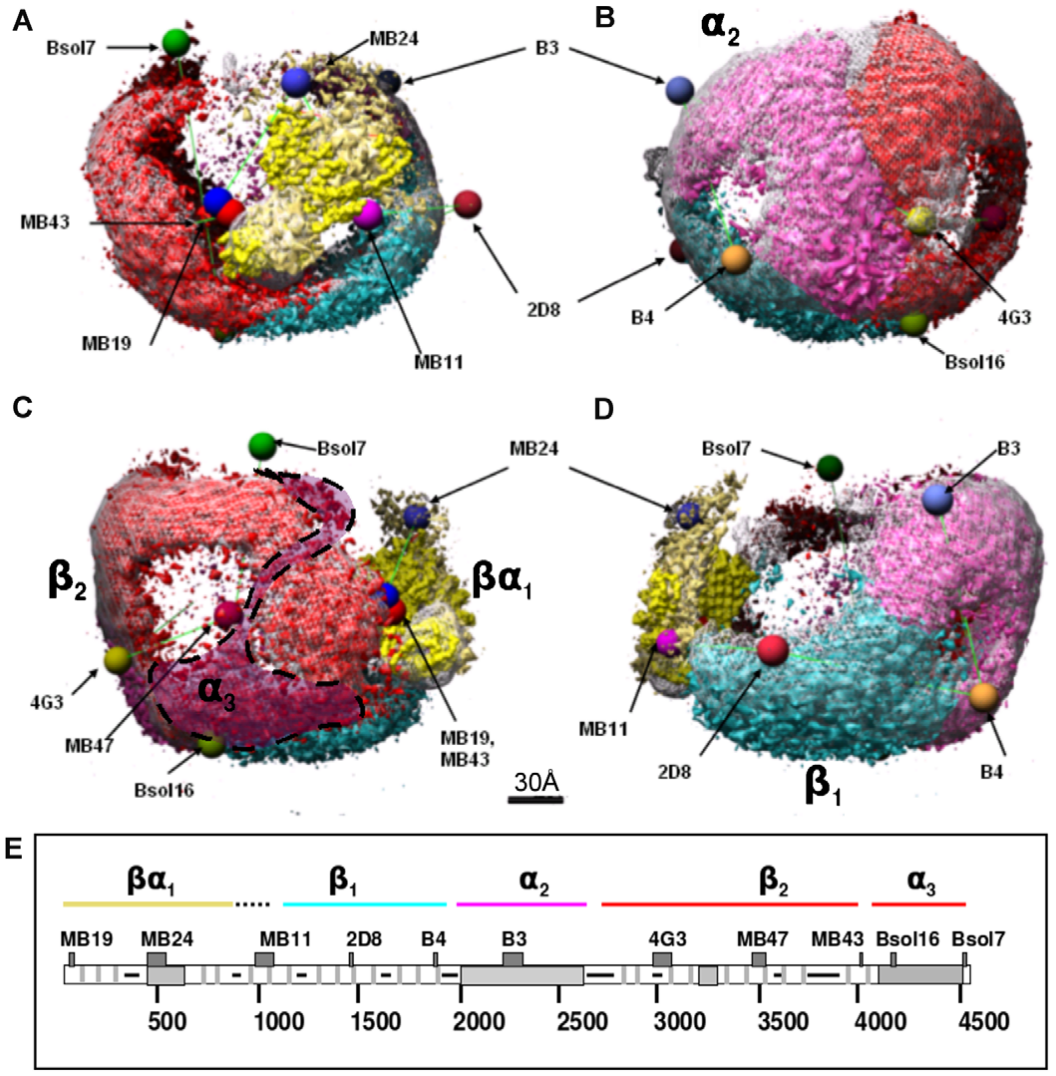
ApoB-100 domains and antibody locations in reconstruction of LDL at 37°C. An LDL particle at 37°C (white mesh isosurface at the threshold of µ+2σ) with different segments (colored), docked lipovitellin (N-terminal amino acids 1–680 shown in yellow), and fitted antibodies' locations. These segments were found automatically by 3D segmentation and their unfiltered versions are shown in different colors at threshold at which the whole reconstructed 3D volume represents a protein mass of 550 kDa. The pentapartite model of apoB-100 is shown in the bottom together with the epitope locations of the antibodies (MB19, 71; MB24, 405–539; MB11, 995–1082; 2D8, 1438–1480; B4, 1854–1878; B3, 2239–2331; 4G3, 2980– 3084; MB47, 3429–3453 and 3507–3523; MB43, 4027–4081; Bsol16, 4154–4189; Bsol7, 4517–4536). Different colors are used for different supra-molecular structures of apoB-100 in all subfigures. The rectangular segment shown with cyan color, between antibody locations MB11, 2D8 and B4, most likely represent the β1 region (approximately from residue 827 to 2000). The segment, shown in magenta, surrounded by antibody locations B3, B4 and 4G3, likely refers to the α2 region (approximately from residue 2075 to 2570). The largest segment surrounded by Bsol7, MB19, Bsol16 and 4G3, shown in red color, appears to be consisting of the β2- (approximately from residue 2571 to 4050) as well as the α3-region (approximately from residue 4050 to 4500). The segmentation tool could not separate the β2- and α3-regions, possibly due to their overlapping locations. However, the docked antibodies clearly point to the possible location of the α3-domain shown shaded and encircled with a dashed line.

The resolution of the current models enabled us to automatically dock the atomic model of the N-terminal 780 amino acids of lipovitellin (23% sequence identity, 40% similarity to the N-terminus of apoB-100) into the 37 °C reconstruction using ADP_EM. The first 260 amino acids (β-barrel) fitted into the protruding knob of the 37°C structure, as shown in **Figure 5**. Just as predicted by other investigators [35], [36] the highest correlation fitting result placed the non-amphipathic α-helical domain (amino acids 270–630) of lipovitellin towards the outside and far from the lipid core of LDL. It also placed the amphipathic β-sheet (amino acids 680–780) towards the inner lipid core. We then used an EMAN 3D segmentation tool on the reconstructed 3D volume of LDL particles at 37°C at an intensity threshold corresponding to the protein mass of apoB-100 (550 kDa). A segment found by this 3D segmentation is supposed to have no connection or only a loose connection with other segments. Four segments were found automatically by 3D segmentation and their unfiltered versions are shown in **Figure 6** **and Videos S1 and S2** using different colors at the threshold at which the whole reconstructed 3D volume represents a protein mass of 550 kDa. The boundaries between the four segments also may correspond to the possible boundaries between the apoB-100 supramolecular structures (NH- βα_1_-β_1_-α_2_-β_2_-α_3-_COOH) [4], [10], [42], with the fitting of the lipovitellin identifying the amino terminal α_1_ domain. The docked lipovitellin was used to infer the location of binding sites for three monoclonal antibodies that have been described [39]. The positions of these three antibodies were then used to fit a triangulation-model given by Chatterton et al. [39] containing an additional 7 antibodies onto the three-dimensional structure. The triangulation-model with additional antibody binding sites that have been mapped to the primary sequence of apoB-100 (**Figure 6**) gave a rough estimation of the physical location for the components of the pentapartite model of apoB-100 (NH- βα_1_-β_1_-α_2_-β_2_-α_3-_COOH) [4], [10]. Thus the triangulation model helped to identify the 4 different protein regions found by segmenting the 37°C structure.

**Figure 7:**
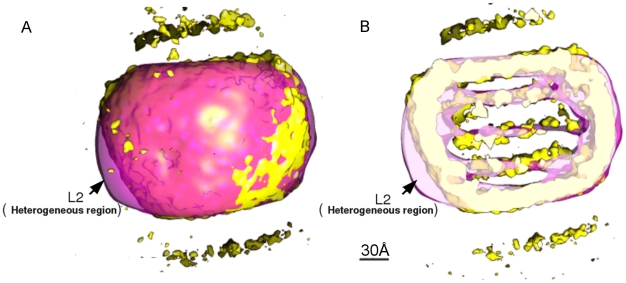
Similarity between 6°C LDL reconstruction and previously published low-resolution structure of LDL-LDLr complex at 4°C. The result of automatic alignment (Align3d in EMAN) of our 6°C LDL reconstruction in yellow with that of LDL in complex with the LDL-receptor (LDL-LDLr) in violet at 4°C (23). The published LDL-LDLr reconstruction (23) was downloaded from the EMdatabank (emdatabank.org accession code EMD-5158). The isosurfaces were generated at a threshold of µ+σ. The view from one side is shown in (a) and the view from the same orientation is shown in (b) as a thick central section from both volumes. Notice that nearly all the regions of volumes of 6°C LDL and LDL-LDLr overlap except the heterogeneous region L2. The 3D volume of 6°C LDL is low-pass filtered to 15 Å resolution and LDL-LDLr is low-pass filtered to 28Å.

**Figure 8:**
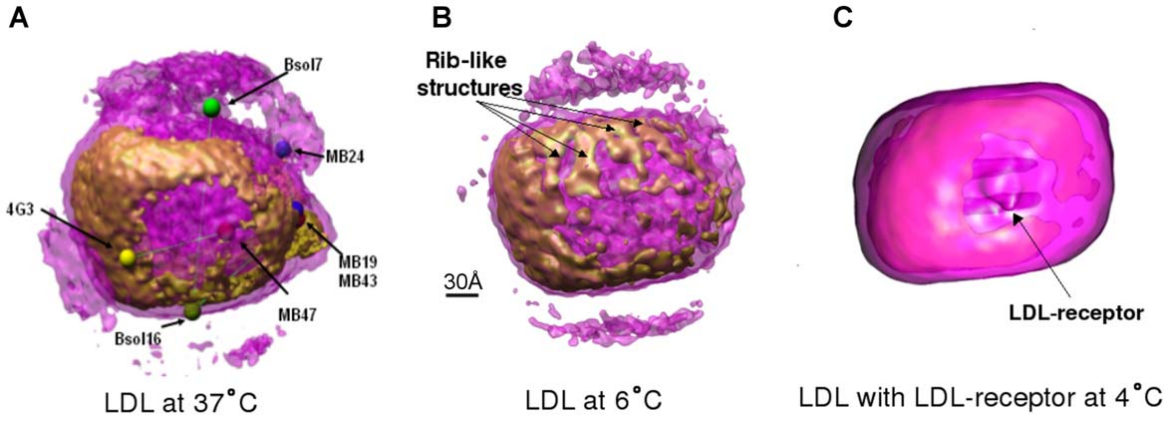
Similar orientation views show LDL-receptor binding site in reconstructions of LDL at 6°C and 37°C and LDL-LDLr complex. The published 3D reconstruction LDL in complex with the LDL-receptor (LDL-LDLr) (23) EMdatabank accession code EMDB-5158. The orientation of the 3D reconstruction of the LDL-LDLr complex was determined by using automatic alignment with 6°C LDL map. For LDL at 37°C and LDL at 6°C the transparent violet iso-surfaces are made at threshold of µ+0.7σ (representing lipid) and opaque brown high density iso-surfaces are made at threshold of µ+3σ (representing rigid structures of protein). For LDL-LDLr complex the transparent violet isosurface is made at threshold of µ+0.25σ and opaque pink high density isosurface is made using a threshold of µ+2.5σ. The 3D volumes of 6°C LDL and 37°C LDL are low-pass filtered to 15Å resolution and 3D map of LDL-LDLr is low-pass filtered to 28Å. In (a) LDL at 37°C is also shown with fitted antibodies locations. The shown antibodies with their epitope locations on the apoB-100 are MB19 (71), MB24 (405–539), 4G3 (2980–3084), MB47 (3429–3453, 3507–3523), MB43 (4027–4081), Bsol16 (4154–4189) and Bsol7 (4517–4536). In (b) the rib like structures in 6°C LDL are indicated by arrows. In (c) the LDL receptor is visible in LDL-LDLr complex. The visible location of LDL-receptor in LDL-LDLr complex corresponds to the region close to MB47 lying in between 4G3 and MB47 mAb locations in LDL at 37°C.

The high intensity regions corresponding to the protein structure in the 3D volumes of LDLs at 6°C and 37°C were computationally checked and confirmed to be rotationally aligned with each other at low resolution. Thus, we could predict the region of the LDL-receptor binding site at both temperatures. To further corroborate this prediction we also aligned our 3D volume of LDL at 6°C (resolution ∼16 Å) with the very recently published 3D volume (resolution 28 Å) of a complex of LDL with the LDL-receptor (LDL-LDLr) at 4°C [23]. The result by an automatic alignment tool (Align3D in EMAN, [29]) is shown in **Figure 7**. It can be seen that most of the regions of the LDL reconstruction at 6°C overlap with the corresponding regions in the LDL-LDLr complex at 4°C. The region that shows partial-overlap is the heterogeneous region L2 found to have high 3D variance in our bootstrap analysis (see **Figure 4**). After automatic alignment we found that the LDL-receptor in the LDL-LDLr complex is located in exactly the same place predicted by fitting the monoclonal antibody triangulation map to our LDL volume. As shown in **Figure 8**, the LDL-receptor lies in the region between the locations of antibodies 4G3, MB47, Bsol16 and Bsol7.

## Discussion

Understanding the molecular structure of LDL particles is challenging due to both methodological issues and the fundamental structural complexity, which is inherent to these particles. Here, we applied cryo-electron microscopy with three dimensional image reconstruction to study the LDL particles at 6°C and 37°C. Because no staining is involved and because particularly low electron doses are used in the image collections (to avoid radiation damage), cryo-EM images are very noisy. Yet, it is important to note that, at present, cryo-EM seems to the best method for the generation of molecular level information on native LDL-particles and apoB-100. This is because no diffracting crystals are available of either LDL particles or apoB-100 for X-ray crystallography [9]. The remarkably good resolution of ∼16 Å achieved in this work with cryo-EM reconstructions using an extensive number of individual LDL particle images and a sophisticated denoising method [25], also points toward the potential of cryo-EM in structural studies of large lipid-protein aggregates, like the various lipoprotein particles. This achieved methodological advancement, however, cannot fully solve the fundamental difficulties caused by the inherent molecular complexity and heterogeneity of the non-covalently associated lipid molecules and apoB-100 present in individual LDL particles. Also the heterogeneous distribution of LDL particles with respect to physical isolation methods, such as density in ultracentrifugation, may increase the potential for bias in the structural analysis. Yet, novel important insights into structure/function and physiology of LDL particles has been gained when applying cryo-EM image processing and 3D reconstruction on LDL particles of the widest density (size) distribution (between 1.006 and 1.063 g/ml) (23). In the present study, we attempted to reduce size heterogeneity by collecting LDL particles within the densities ranging from1.019 to 1.050 g/ml. In future studies, gel filtration separations should be used which would allow still better selection of relatively size-homogenous particles prior to structural analysis.

At low temperature, the overall structure of the LDL particles appears to be semi-discoidal with a protrusion, as has been indicated before in a low resolution cryo-EM study [21], a notion which has also been supported by X-ray crystallography data [43]. The physical explanation for the shape likely relates to the strong non-polar interactions between the CE molecules in the smectic liquid crystal-like phase at the low temperature, which contrasts with the liquid-like organization of CE (and also the minor lipid) molecules in the LDL core at the physiological temperature. At both temperatures, extensive lower-density regions also appear at the surface of the particles (**Figure 3**). These patches on the LDL surface do not seem to have any rigid protein structure (**Figures 3** **,** **4** **,** **5**). It has been suggested that there are such protein-free surface nanodomains consisting mainly of phospholipids with intervening cholesterol and cholesteryl ester molecules [4], [8]. Therefore, at the physiological temperature, the core CEs being in a liquid-like state, the lipid-lipid interactions with an intrinsically self-organizing energy optimum provide a good rationale for the overall spherical shape of the LDL particles.

The lipid nanodomains at the surface of the LDL particles most likely have a significant role in both physiological and pathophysiological processes inherent to LDL metabolism (4). For example, in the circulation cholesteryl ester transfer protein (CETP) is known to attach to these domains [44]. The lipid nanodomains are also important in enzymatic and oxidative modifications of the particles, leading to lipid-lipid interactions between lipoprotein particles. The subsequent fates of such interacting particles, such as particle aggregation and fusion, are the initial processes leading to lipid accumulation in the arterial intima during early atherogenesis [3], [4], [5].

Comparison of the two structures and of their variance allowed us to define two regions within the particle which are most affected by temperature change. In line with previous studies, we see an increase in order at 6°C in the center of the particle [17], [21]. This clearly relates to the phase change of the core lipids. However, the perimeter of the particle also clearly differs. The majority of the well-defined surface at 37°C (visible at a higher threshold in **Figure 3**) probably corresponds to the apoB-100, holding the lipids. However, there is a significant area of the surface that forms a less-well defined density. It is most probably a continuation of the surface phospholipids, devoid of protein. At 6°C, this lipid density is also better defined, which may follow from the stiffening or phase change that occurs for phospholipids at lower temperatures. The apoB-100 protein itself is not known to undergo extensive conformational changes with a shift in temperature, and, hence, it is likely that the differences relate to the lipid rather than the protein.

Segmentation of the well-defined density in the 37°C LDL particle reconstruction along with fitting of a lipovitellin atomic model and comparison to both older antibody mapping studies and the recent structure at 4°C of LDL particles in complex with the LDL receptor all contribute to our current understanding of the organization of the apoB-100 supramolecular structure shown in **Figure 6** [23], [36], [39], [40], [45]. The key differences in our approach were the noise-reduction method applied during the reconstruction and the fitting of the lipovitellin atomic model. These led to a successful higher-resolution 3D reconstruction of native LDL. The presence of other markers such as antibodies that have been attempted earlier may contribute to additional heterogeneity due to conformational flexibility and incomplete labeling, adding to the noise, [22] thereby lowering the resolution of subsequent 3D reconstructions.

The segmentation of the 37°C LDL particle reconstruction did not separate the β2 and α3 regions of the NH- βα_1_-β_1_-α_2_-β_2_-α_3-_COOH pentapeptide, possibly because of their overlapping locations. However, the docked antibodies clearly point to the possible location of the α3 domain (shaded area encircled with a dashed line in **Figure 6**, bottom left, and green in the **Videos S1 and S2**. This interpretation is also supported by the variance computations; on the 3D reconstructions, the areas marked as H2/H3 (**Figure 4**) suggests the presence of a location in apoB-100 with variance which surpasses that of the other surface regions. This location is surrounded by the monoclonal antibody positions Bsol16, 4G3, and MB47 in the C-terminus of apoB-100, and, accordingly, most likely reflects increased mobility in β2 and/or α3 regions. In fact, this interpretation well agrees with previous results [39], which demonstrated that the remaining 11% of apoB-100, i.e., the α3 alpha-helical region, is rather mobile and also controls the visibility of the apoB-100 LDL-receptor binding region during the conversion of VLDL to LDL in the circulating blood plasma.

In the recently reported 3D reconstruction using cryo-electron microscopy data from complexes of LDL and the LDL receptor at 4°C [23], a possibility exists that the selected projection images reflected only LDL particles belonging to a particular subclass. However, the automatic alignment of the 3D volume of the LDL-LDLr complex at 4°C with current 3D LDL volume at 6°C confirmed the correctness of the predicted LDL-receptor binding site as well as the fitting of monoclonal antibody locations (see **Figures 7** and **8**). As expected, the high-variance region L2 (see **Figures 4** and **7**) that has a small non-overlapping region with the LDL-LDRr complex, lies on the α_2_ domain of apoB-100. It fits with earlier models and observations that this major α-helix rich domain is loosely bound to lipids and it can desorb from and reabsorb onto the LDL particle surface [10], [14], [42]. After confirming the location of apoB-100 supra-molecular structures in LDL at both temperatures we tried to find features at high resolution which could give further insight in to the structural details of apoB-100 at different temperatures. One such feature is clearly distinguishable in LDL particles at 6°C at the intensity above the threshold corresponding to a protein mass of 550 kDa, near the LDL-receptor binding site in β2 domain (see **Figure 8**). In the 3D map of LDL at 6°C there are ordered rib-like structures in the region of β2 domain but such ordered rib-like structures are not visible in the corresponding locations at 37°C even though both volumes have the same resolution. We tried different thresholds to highlight such rib-like structures in 3D volume of LDL at 37°C but we could not observe similar features in the whole volume of LDL at 37°C. We could not link them directly to high-resolution secondary-structure details of β2 domain of apoB-100. Generally it has been proposed that the β2 domain is composed of lipid-associated β-sheets [10], [23], [42]. Coronado and Antwerpen [46] reported a decrease in anti-parallel β-sheet content of apoB-100 with an increase in the temperature from 7°C to phase-transition temperature of normal-LDL core (28°C). Such local-conformational changes in apoB-100 could be confined to their own domains, while the major folding of apoB-100 around the lipid core remains the same at 6°C and 37°C. Several studies have tried to link LDL-receptor binding affinity of normal-LDL and TG rich-LDL with apoB-100 confirmation and physical state of the lipid core [16], [17]. The complete explanation and insight of such phenomenon awaits the structure determination of the receptor binding region of apoB-100 in native LDL to an even higher resolution.

The direct benefit of the achieved medium resolution (∼16 Å) structures of lipids and apoB-100 in native LDL particles is that they define clear spatial boundaries for molecular-modeling for the determination of higher resolution features at both temperatures (6°C and 37°C). Even though heterogeneity would always pose a challenge, the 3D variance map of LDL calculated by bootstrap analysis, raises hope that not all the apoB-100 domains are flexible. Some domains are rigid and determining their structural detail could be within reach for the current state-of-the-art cryo-electron microscopy techniques. Currently there are four main approaches in the literature to handle sample heterogeneity with cryo-EM based 3D reconstruction. These approaches are Maximum likelihood methods [48], focused 2D classification [32], sorting using, for instance, multivariate statistical analysis prior to 3D reconstruction [49] and *ab initio* methods [50]. These methods have been shown to work relatively well for two state systems, *i.e.* for sorting out mixed ribosome populations [48] and mixed chaperone populations [51]. The success with even three, let alone four states is very low. Several researchers have used radial profiling from MSA [49] to separate projections from different configurations, and this is also a common approach with icosahedrally-symmetric viruses. For the present study, radial profiling was chosen as one way to approach the heterogeneity in LDL particles, since these particles have a normal distribution due to variable lipid composition and since this affects apoB-100 conformation. Although we have interpreted the reliable areas in the reconstructions as being domains of apoB-100, there is also the possibility that we are looking at averages of different conformations of apoB-100. Unfortunately, the methods currently available do not allow us to distinguish between these two possibilities.

### Conclusions

Due to complexity issues related to the huge molecular size of apoB-100 and to the large number of lipid molecules in the particles, structural and functional studies of these particles pose challenges. Being micellar complexes, the LDL particles share the typical lipoprotein structure with an amphipathic surface monolayer and a hydrophobic lipid core. Since the particles are biologically functional only in this complex state of a non-covalent apoB-100-lipid aggregate, understanding their 3D structure in an aqueous environment is particularly valuable. Using modern cryo-EM techniques with a large number of images of individual LDL particles, we have improved the resolution to ∼16 Å and presented some new information regarding the structure of native LDL particles and apoB-100. Due to the predominance of CE molecules in the particle core, the shape of the LDL particles is temperature-sensitive; at physiological temperatures the particles appear spherical and at lower temperatures discoidal, as has been observed in many laboratories previously. While the present 3D model on the LDL-encircling apoB-100 is still rather crude, it is a step forward towards a coherent experimental determination of the approximate locations of the apparent domains in the pentapartite NH-βα1-β1-α2-β2-α3-COOH model of apoB-100 on the surface of native LDL particles. Albeit several aspects remain to be defined regarding the conformation and function of native apoB-100, particularly regarding molecular interplays between particle lipids and the apoB-100, this study shows the potential of cryo-EM in resolving at least some of the concealed structural details of native LDL particles also at the physiological body temperature.

## Supporting Information

Figure S1 **The radial profiles of reference-free classification-averages and projection from 3D volumes of LDL.** The radial profiles of reference-free class averages indicate that majority of the LDL particles were similar in size. Cross-correlation between radial profiles of reference-free classes and projections were used to further remove images. The radial profiles are shown at sampling-rate of 2.8Å/pixels. (a) Reference-free class-averages of images of LDL at 37°C (b) Projections of 3D volumes of LDL at 37°C. (c) Reference-free class averages of LDL at 6°C obtained using reference free classification. (d) Projections of 3D volumes of LDL at 6°C.
(TIF)

Figure S2 **Resolution estimation by EMAN-eotest tool using Fourier Shell correlation (FSC) for LDL 3D reconstructions.** The resolution at 0.5 FSC is about 15.8 and 15.9 for LDL volume at 37°C (a) and at 6°C (b), respectively.
(TIF)

Video S1 **Video showing LDL at 37°C with protein shell segments and lipid core.** The lipid core is shown in gray and is slightly transparent, while the protein shell segments from 3D reconstruction of 37°C LDL are opaque and colored as in **Figure 6**, except the suggested α3-region that is shown green in the video. The segments shown represent equivalent segments found automatically by 3D segmentation of unfiltered reconstruction of 37°C LDL at a threshold, when the whole reconstructed 3D volume represents a protein mass of 550 kDa. The video was made using UCSC Chimera [47].
(MPEG)

Video S2 **Video showing LDL at 37°C to visualize regions of overlap between protein shell segments and lipid core.** The regions corresponding to automatically found protein shell segments from 3D reconstruction of 37°C LDL are colored as in **Figure 6**, except the suggested α3-region that is shown green in the video. The lipid core is shown opaque and the protein shell segments are slightly transparent.
(MPEG)

Table S1 Results of the automatic fitting of the first 780 residues of the lipovitellin atomic model backbone to the reconstruction of LDL at 37°C using the program ADP_EM [34]. The orientations (Psi, Theta, and Phi) and coordinates (X, Y, and Z) found are shown. The correlation and Z scores of the top 40 solutions were used to calculate the P-values of the top 10 solutions.
(DOC)
