# Supplementary figures and images for: Three-Dimensional cryoEM Reconstruction of Native LDL Particles to 16Å Resolution at Physiological Body Temperature
Source: PLoS One. 2011 May 9;6(5):e18841. doi: 10.1371/journal.pone.0018841 (PMC3090388; doi:10.1371/journal.pone.0018841)

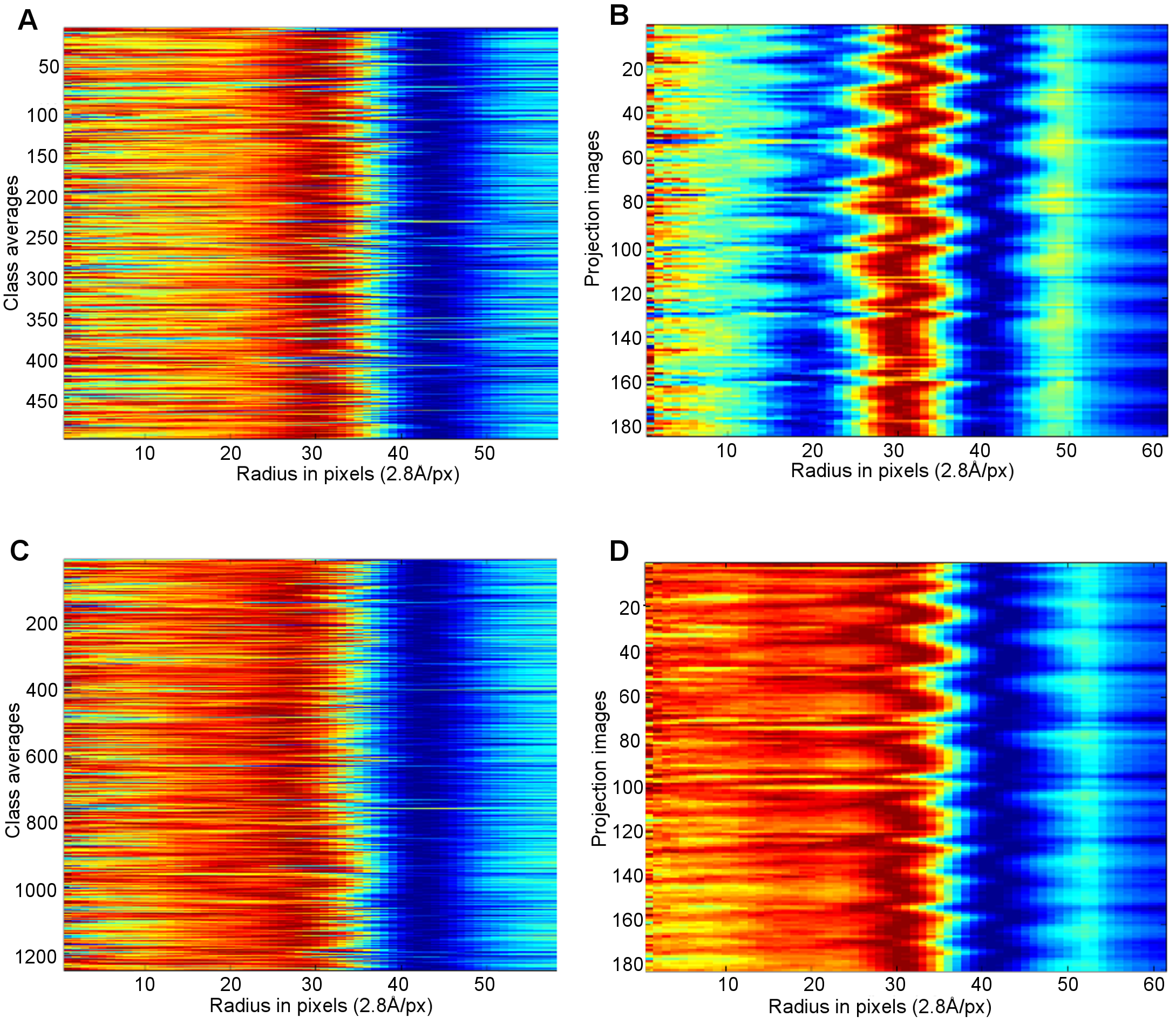

Supplement: Figure S1 — The radial profiles of reference-free classification-averages and projection from 3D volumes of LDL. The radial profiles of reference-free class averages indicate that majority of the LDL particles were similar in size. Cross-correlation between radial profiles of reference-free classes and projections were used to further remove images. The radial profiles are shown at sampling-rate of 2.8Å/pixels. (a) Reference-free class-averages of images of LDL at 37°C (b) Projections of 3D volumes of LDL at 37°C. (c) Reference-free class averages of LDL at 6°C obtained using reference free classification. (d) Projections of 3D volumes of LDL at 6°C. (TIF) [file pone.0018841.s001.tif]

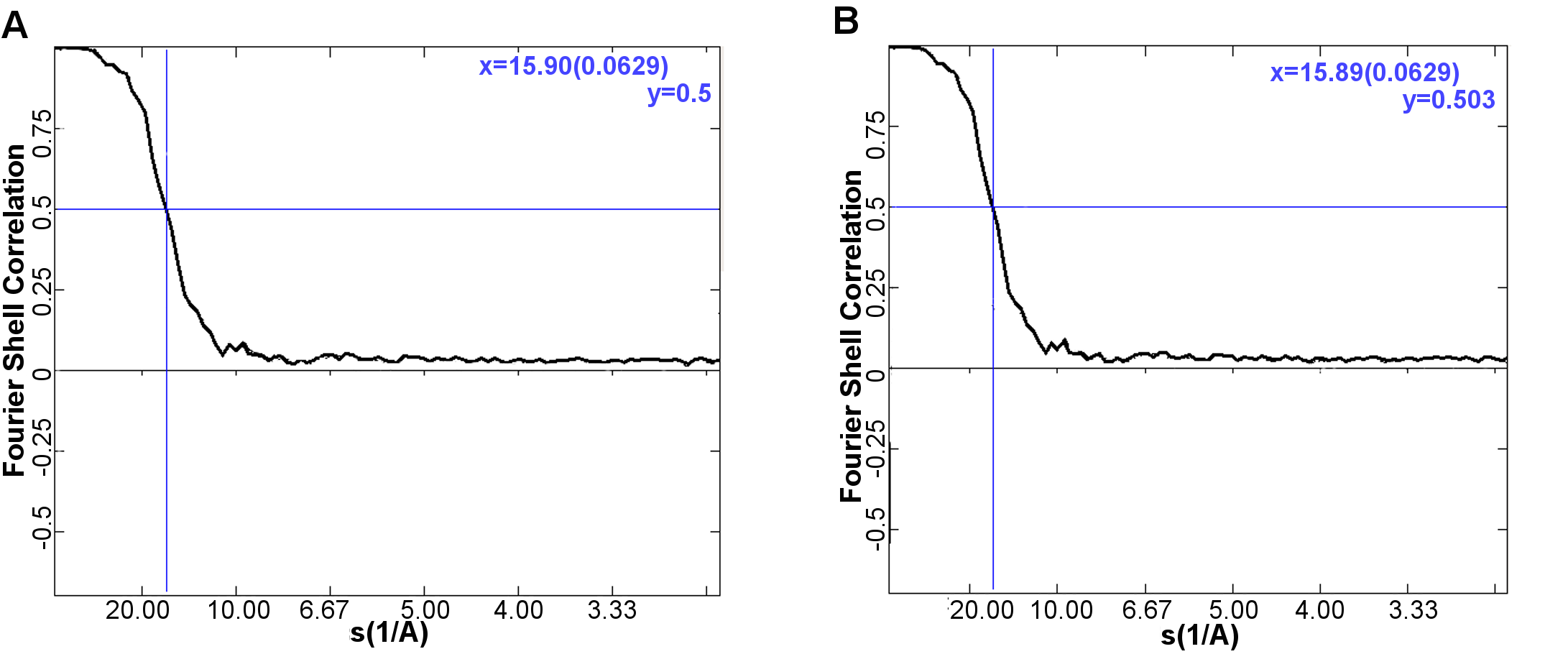

Supplement: Figure S2 — Resolution estimation by EMAN-eotest tool using Fourier Shell correlation (FSC) for LDL 3D reconstructions. The resolution at 0.5 FSC is about 15.8 and 15.9 for LDL volume at 37°C (a) and at 6°C (b), respectively. (TIF) [file pone.0018841.s002.tif]
