# Supplementary material for: Three-Dimensional cryoEM Reconstruction of Native LDL Particles to 16Å Resolution at Physiological Body Temperature
Source: PLoS One. 2011 May 9;6(5):e18841. doi: 10.1371/journal.pone.0018841 (PMC3090388; doi:10.1371/journal.pone.0018841)
Supplement: Table S1 — Results of the automatic fitting of the first 780 residues of the lipovitellin atomic model backbone to the reconstruction of LDL at 37°C using the program ADP_EM [34]. The orientations (Psi, Theta, and Phi) and coordinates (X, Y, and Z) found are shown. The correlation and Z scores of the top 40 solutions were used to calculate the P-values of the top 10 solutions. (DOC) [file pone.0018841.s005.doc]

**Supporting Table S1:**

|  | **Psi** | **Theta** | **Phi** | **X** | **Y** | **Z** | **Corr** | **Z-score** | **P-value** |
| --- | --- | --- | --- | --- | --- | --- | --- | --- | --- |
| **1** | 104.44(24) | 14.63(16) | 243.93(28) | 54.2 | 69.82 | 22.3700 | 0.163 | 2.87937 | 0.00398 |
| **2** | 285.23(7) | 19.67(15) | 73.18(10) | 54.36 | 69.51 | 27.1900 | 0.158 | 2.488947 | 0.01281 |
| **3** | 284.74(7) | 23.96(15) | 158.48(2) | 70.24 | 78.39 | 24.7800 | 0.149 | 1.786186 | 0.07407 |
| **4** | 102.87(24) | 7.63(16) | 200.77(32) | 47.28 | 76.37 | 22.7600 | 0.147 | 1.630016 | 0.10310 |
| **5** | 279.35(8) | 0.00(17) | 4.04(17) | 45.52 | 81.71 | 24.1600 | 0.144 | 1.395763 | 0.16279 |
| **6** | 307.38(5) | 97.23(8) | 106.45(7) | 39.98 | 63.23 | 97.7400 | 0.143 | 1.317678 | 0.18761 |
| **7** | 307.94(5) | 94.50(8) | 60.09(11) | 48.08 | 62.31 | 98.2300 | 0.139 | 1.005339 | 0.31473 |
| **8** | 304.60(5) | 86.23(9) | 82.54(9) | 55.77 | 69.72 | 19.4900 | 0.138 | 0.9272548 | 0.35379 |
| **9** | 284.45(7) | 68.66(11) | 199.35(32) | 45.66 | 80.1 | 39.1200 | 0.136 | 0.7710856 | 0.44066 |
| **10** | 306.75(5) | 133.61(4) | 95.26(8) | 41.99 | 58 | 100.2100 | 0.135 | 0.693001 | 0.48831 |

**Supporting Table S1:** Results of the automatic fitting of the first 780 residues of the lipovitellin atomic model backbone to the reconstruction of LDL at 37°C using the program ADP_EM [34]. The orientations (Psi, Theta, and Phi) and coordinates (X, Y, and Z) found are shown. The correlation and Z scores of the top 40 solutions were used to calculate the P-values of the top 10 solutions.
